# Longitudinal association between parents’ reported vaccination program preferences and children’s actual immunization patterns in Shanghai, China

**DOI:** 10.1186/s12889-025-22253-x

**Published:** 2025-03-14

**Authors:** Zhuoying Huang, Mengdi Ji, Matthew L. Boulton, Jia Ren, Xiaodong Sun, Abram L. Wagner

**Affiliations:** 1 Department of Immunization Program, Shanghai Municipal Centers for Disease Control & Prevention, NO. 1380, West Zhongshan Road, Shanghai, 200336 China; 2 Department of Epidemiology, School of Public Health, University of Michigan, 1415 Washington Heights, Ann Arbor, MI 48109 USA; 3 Department of Internal Medicine, Division of General Medicine, University of Michigan Medical School, 1500 East Medical Center Drive, Ann Arbor, MI 48109 USA

**Keywords:** Vaccination, Immunization programs, Vaccine hesitancy, Parental attitudes, Health knowledge, Attitudes, Practice, China, Pediatrics

## Abstract

**Background:**

As China expands its national immunization program, it is essential to understand parents’ beliefs about pediatric vaccination programs and the translation into actual vaccination decision-making for their children. This study aims to characterize parents pediatric vaccination program preferences and assess the association between parents’ reported vaccination preferences and their children’s vaccination status.

**Methods:**

In a prospective cohort study in Shanghai, China, we linked parents’ survey responses about their preferences for pediatric vaccine programs when the child was ≤ 3 months in 2017 to their children’s immunization records in 2020. We classified parents by their vaccination program preferences through a latent class analysis (LCA). Logistic regression analysis was used to explore the association between immunization patterns and respondents’ LCA results.

**Results:**

The 469 parents were split into four classes: governmental clinic advocates (20%), careful deciders (45%), convenience-focused (19%), and prefer less co-administration (16%). Among the children 66% received combination vaccines, 91% had received at least one imported vaccine, and the average number of office visits by the age of six months was 7.

**Conclusions:**

There were no associations between parents’ reported preference categories and children’s vaccination patterns. The high acceptance of combination vaccines and tolerance of co-administration gives parents choices for vaccination and impart increased confidence in including new vaccines in the vaccination program.

**Supplementary Information:**

The online version contains supplementary material available at 10.1186/s12889-025-22253-x.

## Introduction

China has relatively few routinely recommended vaccines covered in the government-funded Expanded Program on Immunization (EPI) compared to other middle-income countries. The EPI in China provides the following domestically produced vaccines for free to any child which are required for school entry: measles-mumps-rubella combined vaccine (MMR), polio vaccine, diphtheria-tetanus-pertussis combined vaccine (DTP), diphtheria-tetanus combined vaccine (DT), tuberculosis vaccine (BCG), hepatitis A vaccine, hepatitis B vaccine, Japanese Encephalitis vaccine (JEV) and meningococcal vaccine [1]. These EPI vaccines are recommended to be administered from birth to six years of age. Many countries include the *Haemophilus influenzae* type b (Hib) vaccine, pneumococcal conjugate vaccine (PCV), and rotavirus vaccine (RVV) in their EPI program, but those require out-of-pocket payment from parents and are not mandatory in China [2]. Some EPI covered vaccines are also available in an imported, foreign manufactured version that also require parents’ out-of-pocket payment, such as the imported hepatitis A and hepatitis B vaccines, JEV, AC meningitis conjugate vaccine [2]. Additionally, the EPI is expanding sub-nationally in China. For example, the varicella vaccine (VZV) is now recommended and provided for free in several cities including Tianjin, Shanghai, Shenzhen and multiple cities in Jiangsu Province. However, in most other regions in China, it remains a non-EPI vaccine and requires out-of-pocket payment.

The coverage rate in metropolitan areas like Shanghai is particularly high, with 99% coverage for three doses of DTP, 100% of polio, and 92% for the newly added VZV vaccine [3]. Conversely, uptake of non-EPI vaccines is relatively low. Nationally, most non-EPI vaccines have coverage lower than 50% [4], and in Shanghai, coverage varies by vaccine, with uptake at 68% for Hib vaccine but only 13% for PCV [3]. (3) Factors contributing to low uptake likely include a relatively high price for these vaccines and the lack of governmental mandates for receipt [2, 5]. Although less studied, vaccine hesitancy could also impact low uptake [3]. Vaccine hesitancy, defined by the WHO as “delay in acceptance or refusal of vaccination despite the availability of vaccination services,” [6] has been present in China to varying degrees, particularly as new vaccines are introduced into the EPI. Studies have shown that parental vaccine hesitancy is more strongly associated with non-EPI vaccines than with EPI vaccines [3]. Decisions regarding non-EPI vaccines often depend on factors such as availability, cost, and perceived benefits of the vaccine [7]. Additionally, some parents may exhibit general vaccine hesitancy or hold concerns about vaccine safety and efficacy, which can influence their decision-making across both EPI and non-EPI vaccines. Understanding modifiable factors like parents’ preferences toward the vaccination schedules or programs that may induce hesitancy could help inform efforts to increase the uptake of the non-EPI vaccine and raise population-level immunity.

Parents may have substantial concerns about expanding the number of vaccines included in the EPI recommended schedule. Vaccines could be added to the list through co-administering multiple vaccines together in different injections but at the same visit, or by developing combination vaccines, which include multiple vaccine strains in the same vial. A previous survey showed that 64% of the parents in Shanghai were concerned about vaccine co-administrations and 31% expressed reservations about infants receiving too many vaccines by six months of age [8]. Another study revealed substantial concerns from parents about vaccine safety and the pain of multiple injections administered at a single office visit [9]. In a 2017 study of preferences for co-administered vs. combination vaccines, we found that parents in Shanghai were willing to pay $104 to avoid an additional injection at one appointment [10]. Stated preference research in the US found that parents would like to pay around $10 to reduce one injection [11]. These strong reported preferences point to potential difficulties in modifying the vaccine schedule in the future. Combination vaccines are a potential strategy, although they generally come with higher costs and are sometimes more challenging to coordinate with other recommended serial vaccinations [11]. Therefore, investigating parental preferences for childhood vaccination schedule and program settings is crucial to providing needed evidence to maximize vaccine uptake in the face of changing vaccination recommendations and regular additions to the EPI schedule.

Little research from low- and middle-income countries has investigated parents’ preferences around pediatric vaccination programs. In addition, there is a paucity of longitudinal studies globally that explore parents’ reported preferences in the context of their children’s actual vaccination patterns. In order to help address these gaps, in 2017 we conducted a baseline survey studying parents’ preferences for vaccination programs when making vaccination decisions for their children through discrete choice experiments (DCE) in Shanghai, China. Following up on this survey, in 2020 we retrieved the children’s vaccination records to link with their parents’ responses from three years prior. Therefore, this study aims to characterize parents pediatric vaccination program preferences in Shanghai, China. The study also assesses the association between parents’ reported vaccination preferences and their children’s vaccination status.

## Methods

### Study population

This is a prospective cohort study, with the baseline data collected between May and September 2017 and the vaccination records retrieved electronically in the summer of 2020. Details of the baseline survey data are described elsewhere [7]. In summary, all 16 districts of Shanghai (excluding Chongming, a less-populated island district) were included in the study. Each district is composed of 10–36 townships. From these districts, 40 townships were selected based on population size according to the 2010 Census, ensuring a representative geographic distribution. Within each selected township, a public immunization clinic (government-run clinics that provides both mandatory (free) and optional (self-paid) vaccinations. They usually locate in community health centers). was randomly selected, and then a convenience sample of parents of infants younger than three months old attending the clinic was selected. The inclusion criteria were being a parent, grandparent, or guardian of an infant ≤ 3 months, and being ≥ 18 years old. Due to the small sample size of grandparents and other guardians, only parents’ responses were included in the final analysis. The sample size was calculated based on the desire to estimate the association between vaccine hesitancy and vaccine uptake. Based on the pilot study from 2014 in Shanghai, we expected a pneumococcal vaccination uptake to be 13.9% and 5.5% among parents without and with vaccine hesitancy, respectively. With an intra-cluster coefficient (ICC) of 0.02389 (also estimated from the pilot study), an alpha of 0.05, and a power of 0.80, at least 647 participants needed to be recruited.

### Vaccination pattern

We abstracted children’s immunization records from the Shanghai Immunization Program Information System in August 2020. The records include the children’s date of birth and information on vaccination, including the date of administration, manufacturer, and whether administered as a combination vaccine. We examined receipt of a quadrivalent combination vaccine (DTap-Hib), a pentavalent combination vaccine (DTap-Hib-Polio), and at least one dose of foreign manufactured vaccine. We also derived one categorical variable: number of office visits by the age of six months old of children (5 visits, 6 visits, 7visits, 8 visits, and 9–13 visits).

### Vaccine hesitancy

Parents’ vaccine hesitancy was measured through a 10-item scale developed by the WHO SAGE Working Group on Vaccines [6]. The scale was developed through systemic peer-reviewed literatures and validated through a global pilot test [6]. Descriptive results from the scale are presented elsewhere [7]. The scale contains 10 items with 5-point Likert options (1 = strongly disagree, 2 = disagree, 3 = neither agree nor disagree, 4 = agree, and 5 = strongly agree). We dichotomized the scale outcome as vaccine-hesitant respondents and not vaccine-hesitant respondents in a validated way for the ease of further analysis. In brief, we summed the questions so that the scale ranged in possibility from 10 to 50 and then dichotomized at 25, with a higher number representing vaccine-hesitant and a lower number representing non-vaccine hesitant.

### Covariates

Other sociodemographic variables are used as control variables in the multivariable analysis, including the gender of the child, the association of the participants to the child (mother, father, and other), monthly household income, education, and residency. Residency refers to the participant’s official residency, including locals (official urban residency within Shanghai city), urban non-locals (urban residency outside of Shanghai), and rural non-locals (rural residency outside of Shanghai). To note, non-local residents may be restricted from obtaining certain governmental benefits [12]. However, the EPI vaccines are offered free to the public regardless of their residency status.

### Latent class analysis and parents’ classification

The discrete choice experiment conducted in 2017 measured the relative parental preferences for five vaccination attributes [10]: (1) waiting time at clinic (Duration parents spend waiting for vaccination services), (2) vaccination site: (3) governmental clinic/private clinic, (4) the number of visits, co-administration, and (5) costs. Briefly, we assessed willingness to pay for changes in attribute level in a previous analysis [10]. In this study, we classified individuals by vaccination preferences within an exploratory Latent Class Analysis (LCA). LCA can identify unobserved subgroups within a population based on their response patterns or characteristics, which may not be directly measurable. A sensitivity analysis was also conducted to test different numbers of classes (Supplemental Tables). Classes were selected based on the ASkaike Information Criterion (AIC) and Bayesian Information Criterion (BIC) model statistics, with smaller values indicating better model fit.

### Statistical analysis

Parents’ preference categories are classified by latent class analysis. We then linked these preferences to children’s vaccine receipt by linking the vaccination records retrieved from the IIS in the summer of 2020. The associations between the outcome variables—children’s vaccination patterns (receipt of the quadrivalent combination vaccine (DTap-Hib), pentavalent combination vaccine (DTap-Hib-Polio), at least one dose of a foreign-manufactured vaccine (imported vaccine that requires out-of-pocket payment), and the number of office visits—and the independent variable, respondents’ LCA classifications derived through the LCA analysis as previously described, were assessed using multivariable logistic regression, controlling for sociodemographic characteristics. Odds ratios (OR) and 95% of confidence intervals (CI) were reported. Grandparents were excluded from the analysis due to small sample sizes. Sampling weights were used in the analysis. Given the complex sampling scheme and in order to generalize to the population of parents of young infants in Shanghai, we used inverse probability of selection for each participant to derive the sampling weights. The data were analyzed in SAS version 9.4 (SAS Institute, Cary, NC).

## Results

In 2017, 590 participants completed a survey on preferences for vaccination program attributes, and the vaccine records were accessed in 2020 (Table 1). After removing children who moved out of Shanghai by the time the vaccination records were retrieved, 469 (79%) participants remained in the cohort. About two-thirds (64%) were mothers of the children, with the remainder comprising fathers (31%) and grandparents (5%). About half (55%) were locals, with the remainder being rural non-locals (31%) and urban non-locals (14%). The majority of the participants reported they were not vaccine hesitant (81%) and most (85%) had some college education (vocational school, bachelor’s degree and higher).

**Table 1:** Demographic characteristics of study population, Shanghai, 2017–2020 (*N* = 469)

	Count (weighted % ± SE)
Gender of child	
Female	214 (46% ± 3%)
Male	240 (54% ± 3%)
Association to child	
Mother	309 (64% ± 3%)
Father	144 (31% ± 3%)
Other / Grandparent	16 (5% ± 2%)
Residency	
Locals	242 (55% ± 3%)
Urban non-locals	78 (14% ± 2%)
Rural non-locals	144 (31% ± 3%)
Education	
High school or less	61 (15% ± 2%)
Some college or more	402 (85% ± 2%)
Income	
<7,500 RMB (1,028 USD)	147 (36% ± 3%)
≥7,500 RMB	311 (64% ± 3%)
Vaccine hesitancy	
Hesitant	79 (19% ± 3%)
Not hesitant	345 (81% ± 3%)

Table 2 shows children’s vaccination status from 2017 to 2020. More than half of the children received the combination vaccine, either the DTap-Hib combination (quadrivalent) (42%) or the DTap-Hib-Polio combination (pentavalent) (24%). In addition, 91% of the children received at least one dose of an imported vaccine. By the age of 6 months old, about one-third of the children had 6 office visits (35%), one-third of 7 visits (33%), 12% had less than 5 visits; only 7% had 9–13 office visits.

**Table 2:** Children’s vaccination outcomes, Shanghai, 2017–2020 (*N* = 469)

	Count (weighted % ± standard error)
Received quadrivalent vaccine (DTap-Hib)	205 (42% ± 3%)
Received pentavalent vaccine (DTap-Hib-Polio)	104 (24% ± 3%)
Received at least one foreign vaccine*	423 (91% ± 2%)
Number of office visits < 6 months of age	
≤ 5 visits	49 (12% ± 2%)
6 visits	157 (35% ± 3%)
7 visits	162 (33% ± 3%)
8 visits	63 (13% ± 2%)
9–13 visits	38 (7% ± 1%)

^*^ HBV, HAV, Hib, PCV^13^, rotavirus vaccine, quadrivalent influenza vaccine, ACWY meningococcal conjugate vaccine, and pentavalent vaccine are available as imported, foreign−manufactured versions

Parents’ classification and its association with the children’s vaccination patterns are shown in Table 3. Parents were categorized into four classes by their predominant vaccination program preferences: Governmental clinics advocates (20%) who prefer public immunization clinics to private ones; careful deciders (45%) who weigh all the factors related to their vaccine decision-making; convenience-focused (19%) respondents who prioritize fewer office visits for vaccination; and parents who prefer fewer vaccines co-administered (16%) per visit. Sensitivity analyses of classifying parents into 2–3, 5–6 classes are shown in Appendix Tables 1, 2, 3 and 4. Generally, there was no association between parents’ classification and their children’s vaccination pattern. However, within the null results, we observed some suggestive trends. For example, compared to the careful deciders, parents in the other three categories expressed higher acceptance of the quadrivalent combination vaccine and a preference for fewer than eight office visits, even among convenience-focused respondents (OR: 0.70, 95% CI: 0.46–1.05). Additionally, compared to careful deciders, only convenience-focused respondents showed a higher acceptance of imported vaccines (OR: 1.45, 95% CI: 0.92–2.30). These trends may indicate potential patterns that provide further exploration in future studies.

**Table 3:** Associations between parents’ latent classifications and children’s vaccination patterns: results from multivariable logistic regression analysis, adjusting for sociodemographic variables in Shanghai, China, 2017–2020 (*N* = 469)

	Count (weighted %)	Receipt of a quadrivalent vaccine	Receipt of a pentavalent vaccine	Receipt of any imported vaccine	≥ 8 Office visits before age of 6 months
Governmental clinic advocates	91 (20%)	1.02 (0.73, 1.44)	0.98 (0.66, 1.44)	0.83 (0.57, 1.20)	0.99 (0.67, 1.48)
Careful deciders	205 (45%)	ref	ref	ref	ref
Convenience focused	87 (19%)	1.23 (0.91, 1.66)	1.12 (0.77, 1.61)	1.45 (0.92, 2.30)	0.70 (0.46, 1.05)
Prefer less co-administration	73 (16%)	1.10 (0.77, 1.57)	0.99 (0.64, 1.52)	0.98 (0.66, 1.44)	0.93 (0.62, 1.39)

**Table 4:** Association of receiving quadrivalent (DTap + Hib) vaccine and demographic characteristics in Shanghai, China, *N* = 469, 2017–2020

	Percent with quadrivalent vaccine	Odds ratio for receipt of quadrivalent vaccine (95% CI)	*P*-value
Gender of child			0.99
Female	95/214 (44%)	ref	
Male	101/240 (42%)	1.00 (0.57, 1.73)	
Association to child			0.23
Mother	137/309 (44%)	ref	
Father	59/144 (41%)	0.89 (0.49, 1.61)	
Residency			0.02
Locals	133/242 (52%)	ref	
Urban non-locals	31/78 (40%)	0.70 (0.33, 1.49)	
Rural non-locals	37/144 (26%)	0.34 (0.17, 0.71)	
Education			0.97
High school or less	16/61 (26%)	ref	
Some college or more	185/402 (46%)	0.98 (0.34, 2.80)	
Monthly Income			0.07
< 7,499 RMB (1,028 USD)	45/147 (31%)	ref	
>7,500 RMB	153/311 (49%)	1.90 (0.94, 3.82)	
Vaccine hesitancy			0.74
Hesitant	30/79 (38%)	0.88 (0.42, 1.86)	
Not hesitant	154/345 (45%)	ref	

**Table 5:** Association of receiving pentavalent (DTap + Hib + Polio) vaccine and demographic characteristics in Shanghai, China, *N* = 469, 2017–2020

	Percent with pentavalent vaccine	Odds ratio for receipt of pentavalent vaccine (95% CI)	*P*-value
Gender of child			0.60
Female	50/214 (23%)	ref	
Male	48/240 (20%)	0.84 (0.43, 1.63)	
Association to child			0.04
Mother	66/309 (21%)	ref	
Father	32/144 (22%)	0.97 (0.49, 1.94)	
Residency			0.0002
Locals	82/242 (34%)	ref	
Urban non-locals	12/78 (15%)	0.26 (0.11, 0.63)	
Rural non-locals	9/144 (6%)	0.18 (0.06, 0.48)	
Education			0.32
High school or less	3/61 (5%)	ref	
Some college or more	100/402 (25%)	2.45 (0.42, 14.33)	
Income			0.34
< 7,499 RMB (1,028 USD)	17/147 (12%)	ref	
>7,500 RMB	86/311 (28%)	1.61 (0.61, 4.26)	
Vaccine hesitancy			0.56
Hesitant	17/79 (22%)	1.30 (0.54, 3.14)	
Not hesitant	78/345 (23%)	ref	

**Table 6:** Association of receiving at least one dose of foreign vaccine and demographic characteristics in Shanghai, China, *N* = 469, 2017–2020

	Percent with pentavalent vaccine	Odds ratio for receipt of pentavalent vaccine (95% CI)	*P*-value
Gender of child			0.42
Female	192/214 (90%)	ref	
Male	217/240 (90%)	0.70 (0.29, 1.69)	
Association to child			< 0.0001
Mother	279/309 (90%)	ref	
Father	130/144 (90%)	0.57 (0.23, 1.41)	
Residency			0.29
Locals	226/242 (93%)	ref	
Urban non-locals	69/78 (88%)	0.74 (0.26, 2.11)	
Rural non-locals	124/144 (6%)	0.36 (0.10, 1.33)	
Education			0.29
High school or less	54/61 (89%)	ref	
Some college or more	364/402 (91%)	0.38 (0.06, 2.29)	
Income			0.94
< 7,499 RMB (1,028 USD)	124/147 (84%)	ref	
>7,500 RMB	290/311 (93%)	1.04 (0.35, 3.09)	
Vaccine hesitancy			0.002
Hesitant	64/79 (81%)	0.24 (0.10, 0.59)	
Not hesitant	320/345 (93%)	ref	

Tables 4, 5, 6 and 7 present the associations between children’s vaccination patterns and socioeconomic status (SES). Our analysis revealed significant associations between residency, income, parental association, and vaccine hesitancy with vaccine uptake for the quadrivalent and pentavalent vaccines, as well as receipt of at least one dose of a foreign-manufactured vaccine.

**Table 7:** Association of number of office visits before age of 6 months and demographic characteristics in Shanghai, China, *N* = 469, 2017–2020

	Percent with > = 8 office visits	Odds ratio for receipt of pentavalent vaccine (95% CI)	*P*-value
Gender of child			0.28
Female	52/214 (24%)	ref	
Male	45/240 (19%)	0.69 (0.35, 1.36)	
Association to child			0.53
Mother	66/309 (21%)	ref	
Father	29/144 (20%)	0.75 (0.38, 1.48)	
Residency			0.11
Locals	60/242 (25%)	ref	
Urban non-locals	15/78 (19%)	0.57 (0.23, 1.42)	
Rural non-locals	25/144 (17%)	0.41 (0.17, 0.98)	
Education			0.60
High school or less	87/402 (22%)	ref	
Some college or more	13/61 (21%)	0.71 (0.20, 2.50)	
Income			0.73
< 7,499 RMB (1,028 USD)	71/311 (23%)	ref	
> 7,500 RMB	28/147 (19%)	0.87 (0.38, 1.98)	
Vaccine hesitancy			0.69
Hesitant	18/ 79(23%)	0.84 (0.38, 1.89)	
Not hesitant	77/345 (22%)	ref	

### Quadrivalent vaccine

Rural non-local residents were significantly less likely to vaccinate their children compared to local residents (OR = 0.34, 95% CI = 0.17–0.71), indicating a 66% lower likelihood of acceptance. Urban non-locals also had lower odds of vaccinating their children compared to local residents (OR = 0.70, 95% CI = 0.33–1.49), though this result was not statistically significant. Families with a monthly income greater than 7,500 RMB (1,028 USD) were more likely to accept the quadrivalent vaccine compared to those earning less (OR = 1.90, 95% CI = 0.94–3.82), though this association approached but did not reach statistical significance (*P* = 0.07).

### Pentavalent vaccine

Residency demonstrated a strong association. Urban non-locals were 74% less likely (OR = 0.26, 95% CI = 0.11–0.63), and rural non-locals were 82% less likely (OR = 0.18, 95% CI = 0.06–0.48) to vaccinate their children compared to local residents. The parental association (mother vs. father) was not significantly related to pentavalent vaccine uptake (OR = 0.97, 95% CI = 0.49–1.94).

### At least one dose of a foreign-manufactured vaccine

Vaccine hesitancy was a key factor. Vaccine-hesitant parents were significantly less likely to have their children receive an imported vaccine compared to non-hesitant parents (OR = 0.24, 95% CI = 0.10–0.59), indicating a 76% reduced likelihood of uptake. The parental association (mother vs. father) did not show a relationship with foreign vaccine uptake (OR = 0.57, 95% CI = 0.23–1.41).

### Number of office visits

No associations were found between the number of office visits and SES.

## Discussion

In this longitudinal study, we surveyed 590 parents in 2017 about their preferences for pediatric vaccination schedules and programs, then retrieved and linked their children’s vaccination records to investigate the association between parents’ reported preferences and their children’s actual vaccination patterns. Overall, parents demonstrated high acceptance of imported vaccines (91%) and moderate acceptance of combination vaccines (42% for DTap-Hib and 24% for DTap-Hib-Polio). Using Latent Class Analysis (LCA), parents were classified into four categories: governmental clinic advocates (20%), careful deciders (45%), convenience-focused (19%), and those preferring fewer co-administered vaccines (16%). No associations were found between parents’ classifications and their children’s vaccination patterns. However, significant associations were observed between parents’ vaccine hesitancy, socioeconomic factors—such as residency, monthly income, and parental association—and children’s vaccination patterns.

Understanding vaccine decision-making in China has become increasingly important as new vaccines enter the market and in the shadow of the unprecedent rollout of the COVID-19 vaccine. Few prospective cohort studies exist to examine the association between parents’ preferences and their children’s actual immunization patterns. Our study addresses this gap by longitudinally linking the children’s vaccination records with their parents’ attitudes toward vaccine programs. We characterized parents into four groups based on programmatic and vaccination preferences: governmental clinic advocates, careful deciders, convenience-focused respondents, and parents who prefer less co-administration.

Overall, we found no association between parents’ classification and their children’s vaccination status. Our finding of a lack of association between parental preference classification and their children’s vaccination status is seemingly contrary to what we found in the 2017 baseline survey, where the parents express a strong aversion to co-administration [10]. Results from that analysis implied that parents might be more accepting of combination vaccines and might otherwise delay vaccination if co-administration is the only alternative. The null results found here have several explanations. There could be a gap between parents’ belief and their practical options. Stated preference studies, such as were used in our LCA, are by nature hypothetical and that may limit generalizability to a real-world scenario.

Importantly, the null association does not undermine the value of the LCA approach. Rather, it highlights the complexities of translating parental preferences into actionable predictors of behavior. This null association demonstrated that while LCA can identify latent subgroups based on survey responses, further work is needed to understand how these groups translate preferences into vaccination behaviors. Such insights are critical for designing effective vaccination strategies that address not only reported concerns but also practical barriers.

The results may also indicate that parents’ concern about co-administration might be overestimated. This may be due to difficulties measuring concern on this dimension. A systematic review found that providers may overestimate parents’ concerns about co-administration, leading providers to postpone recommended vaccinations, resulting in extra visits and delayed vaccination [13]. A 2011 study found that although parents might be concerned about too much pain, discomfort, and the possibility of “overworking” their child’s immune system with multiple injections, they still wanted to ensure that their child received all the necessary vaccines [14]. Our results again confirmed that co-administration is tolerated by parents in actual practice. In addition, the high acceptance of combination vaccines in our study indicates that future development of more combination vaccines may help increase vaccine uptake. Combination vaccines represent a potential strategy to mitigate the number of visits for vaccination while still providing the same level of immune protection. That said, combination vaccines are relatively high cost, and parents have expressed concerns about the immune overload of the combination vaccine [15]. The apparent higher levels of parental tolerance or even acceptability of vaccine co-administration and combination vaccines potentially provide more options for parents (and vaccine manufacturers) and to help ensure timely childhood vaccination.

Mandatory vaccination, such as requiring vaccines for school, might be another reason for the similar vaccination patterns among different parental preference groups. Previous studies have shown that even if parents are vaccine-hesitant, they are still willing to accept governmentally mandated vaccines based on the recommended schedule [3]. Importantly, there is documented widespread and consistent support for mandatory vaccines in China [16] as evidenced in this study by the high levels of vaccine uptake across the four different parental preference groups. Mandatory vaccination requires a reliable supply of safe and effective vaccines, which may, in turn, strengthen the oversight role of the government in ensuring vaccine safety and effectiveness [17]. Our findings study also indicate a high acceptance of the imported vaccines. This may be partially due to several vaccine scandals of domestic manufactured vaccines, including the 2018 Shandong vaccine crisis [18]. The fallout from this resulted in decreased public trust in domestically produced vaccines, and greater parental hesitancy towards the domestic vaccine which increased from 31 to 83% two weeks after the crisis in 2018 [18, 19]. Despite this, vaccine hesitancy appears to have had a limited impact on China’s infant immunization rate which is above 95% in 2018, according to the WHO surveillance data [20]. This may be partially explained by parents deciding to use imported rather than domestically produced vaccines for their children which is consistent with the findings of our study, and likely related to high compliance with the Chinese government’s mandatory vaccine policy.

Across all vaccination patterns, we did observe disparities in certain vaccinations. Parents’ residency and family income were associated with parents’ choice of combination vaccine and imported vaccine. This might be partially due to the higher cost of combination vaccines (domestic quadrivalent $54/dose, four doses needed, imported pentavalent $86/dose, four doses needed) compared to the single domestic manufactured vaccine, which can be vaccinated for free. Previous studies also found similar patterns that lower coverage of non-EPI vaccines among non-locals compared to locals [3]. As China rapidly urbanizes, more and more families move into cities. Although vaccine services are offered to all residents regardless of residency status, other factors may affect the vaccination decision-making for these new immigrants. For example, a study of migrant children in a city near Shanghai found that larger family size, shorter living duration, and lower family income are associated with a lower non-EPI vaccination rate [21]. As cities expand and new immigrants rush into cities, policies for timely vaccination schedules and benefits education, support for vaccination service navigation, and make-up for overdue vaccines are needed.

### Strength and limitations

A strength of this study is the longitudinal design. We surveyed parents in 2017, about their preferences toward hypothetical vaccination programs and then retrieved children’s vaccination records in 2020 to ascertain how children’s vaccination status corresponded with parents’ reported preferences. To the authors’ knowledge, very few cohort studies like ours have been performed. In addition, our sample was drawn from almost all districts from Shanghai, except one. This study also has some limitations. We used a convenience sample from the governmental clinics and selected parents might favor government clinics more or may have different attitudes toward vaccination programs compared to parents who use private clinics or who do not go to clinics. This selection bias may reduce the generalizability of our findings. Additionally, our cohort was relatively small sample size compared to Shanghai’s population of 22 million residents, which may limit the generalizability of the findings to the broader population. The absence of age data is a key limitation, as age is widely recognized as a critical predictor of health-related outcomes and a common confounder in health behavior research. This omission prevents us from controlling for potential age-related differences that might influence our findings and limits the generalizability of our results. Future studies should incorporate age to enable a more comprehensive analysis of its moderating effects on health behaviors. In addition, we excluded 216 children who had moved out of Shanghai when we retrieved their vaccination records. This may also bias the study since a large proportion of those lost to follow-up (moved out of Shanghai) were disproportionately non-locals.

## Conclusion

We characterized parents in Shanghai, China, by their reported vaccination program preferences and found no associations with their children’s actual vaccination status. There was high acceptance of combination vaccines and tolerance of multiple injections (i.e., co-administration) which give parents more options and lends support for introducing new vaccines into EPI in the future.

### Practical recommendation

To ensure timely and equitable childhood vaccination, promoting affordable combination vaccines is crucial, as they reduce office visits while maintaining coverage. Public health campaigns should address vaccine hesitancy by emphasizing the safety and efficacy of domestic vaccines, rebuilding trust after past scandals, and addressing misconceptions about immune overload from co-administration. Targeted support for migrant families, including clear vaccination guidance, service navigation assistance, and catch-up vaccination programs, is needed to overcome barriers faced by non-locals. Additionally, maintaining and strengthening mandatory vaccination policies, supported by a reliable supply of safe and effective vaccines, will help sustain high immunization rates and public confidence in the immunization system.

## Electronic supplementary material

Below is the link to the electronic supplementary material.

Supplementary Material 1

## Data Availability

Data are available upon reasonable request from the corresponding author.
