# Supplementary material for: Longitudinal association between parents’ reported vaccination program preferences and children’s actual immunization patterns in Shanghai, China
Source: BMC Public Health. 2025 Mar 14;25:999. doi: 10.1186/s12889-025-22253-x (PMC11907872; doi:10.1186/s12889-025-22253-x)
Supplement: Supplementary file 1 — Supplementary Material 1 [file 12889_2025_22253_MOESM1_ESM.docx]

Sensitivity analysis

Supplementary Table 1: Parents were classified into two groups by their vaccination program preferences.

|  |  | Prefer governmental clinics | Careful deciders |
| --- | --- | --- | --- |
|  |  | 15% | 85% |
| Location | Government clinic | 100% | 45% |
|  | Private clinic | 0% | 55% |
| Waiting time | 20 min | 100% | 43% |
|  | 40 min | 0% | 25% |
|  | 60 min | 0% | 14% |
|  | 90 min | 0% | 18% |
| Number of visits | 3 visits | 100% | 33% |
|  | 5 visits | 0% | 25% |
|  | 7 visits | 0% | 24% |
|  | 8 visits | 0% | 18% |
| Cost | 100 RMB | 0% | 25% |
|  | 200 RMB | 100% | 19% |
|  | 400 RMB | 0% | 29% |
|  | 800 RMB | 0% | 27% |
| Number co-administered injections | 1 shot | 0% | 35% |
|  | 2 shots | 0% | 24% |
|  | 3 shots | 0% | 32% |
|  | 4 shots | 100% | 9% |

Supplementary Table 2: Parents were classified into three groups by their vaccination program preferences.

|  |  | Prefer governmental clinics | Careful deciders | Convenience focused |
| --- | --- | --- | --- | --- |
|  |  | 19% | 62% | 18% |
| Location | Government clinic | 100% | 55% | 0% |
|  | Private clinic | 0% | 45% | 100% |
| Waiting time | 20 min | 88% | 47% | 27% |
|  | 40 min | 0% | 24% | 36% |
|  | 60 min | 0% | 19% | 0% |
|  | 90 min | 12% | 10% | 37% |
| Number of visits | 3 visits | 79% | 15% | 100% |
|  | 5 visits | 21% | 27% | 0% |
|  | 7 visits | 0% | 33% | 0% |
|  | 8 visits | 0% | 25% | 0% |
| Cost | 100 RMB | 0% | 34% | 0% |
|  | 200 RMB | 100% | 19% | 0% |
|  | 400 RMB | 0% | 29% | 36% |
|  | 800 RMB | 0% | 18% | 64% |
| Number co-administered injections | 1 shot | 0% | 48% | 0% |
|  | 2 shots | 21% | 16% | 36% |
|  | 3 shots | 0% | 24% | 64% |
|  | 4 shots | 79% | 12% | 0% |

Supplementary Table 3: Parents were classified into four groups by their vaccination program preferences.

|  |  | Prefer governmental clinics | Careful deciders | Convenience focused | Less co-administration |
| --- | --- | --- | --- | --- | --- |
|  |  | 20% | 45% | 19% | 16% |
| Location | Government clinic | 100% | 56% | 0% | 53% |
|  | Private clinic | 0% | 44% | 100% | 47% |
| Waiting time | 20 min | 87% | 57% | 27% | 21% |
|  | 40 min | 0% | 16% | 37% | 47% |
|  | 60 min | 0% | 15% | 0% | 32% |
|  | 90 min | 13% | 13% | 36% | 0% |
| How many visits | 3 visits | 77% | 0% | 100% | 53% |
|  | 5 visits | 23% | 37% | 0% | 0% |
|  | 7 visits | 0% | 29% | 0% | 47% |
|  | 8 visits | 0% | 34% | 0% | 0% |
| Cost | 100 RMB | 0% | 28% | 0% | 53% |
|  | 200 RMB | 100% | 8% | 0% | 47% |
|  | 400 RMB | 0% | 39% | 37% | 0% |
|  | 800 RMB | 0% | 24% | 63% | 0% |
| Number co-administered injections | 1 shot | 0% | 30% | 0% | 100% |
|  | 2 shots | 23% | 20% | 37% | 0% |
|  | 3 shots | 0% | 33% | 63% | 0% |
|  | 4 shots | 77% | 17% | 0% | 0% |

Supplementary Table 4: Parents were classified into five groups by their vaccination program preferences.

|  |  | Prefer governmental clinics | Careful deciders | Convenience focused | Less co-administration | Cost-sensitive |
| --- | --- | --- | --- | --- | --- | --- |
|  |  | 20% | 39% | 19% | 8% | 15% |
| Location | Government clinic | 100% | 50% | 0% | 0% | 100% |
|  | Private clinic | 0% | 50% | 100% | 100% | 0% |
| Waiting time | 20 min | 87% | 66% | 27% | 0% | 23% |
|  | 40 min | 0% | 2% | 37% | 100% | 42% |
|  | 60 min | 0% | 17% | 0% | 0% | 35% |
|  | 90 min | 13% | 15% | 36% | 0% | 0% |
| How many visits | 3 visits | 77% | 0% | 100% | 0% | 58% |
|  | 5 visits | 23% | 27% | 0% | 0% | 42% |
|  | 7 visits | 0% | 33% | 0% | 100% | 0% |
|  | 8 visits | 0% | 40% | 0% | 0% | 0% |
| Cost | 100 RMB | 0% | 17% | 0% | 0% | 100% |
|  | 200 RMB | 100% | 9% | 0% | 100% | 0% |
|  | 400 RMB | 0% | 46% | 37% | 0% | 0% |
|  | 800 RMB | 0% | 28% | 63% | 0% | 0% |
| Number co-administered injections | 1 shot | 0% | 34% | 0% | 100% | 58% |
|  | 2 shots | 23% | 23% | 37% | 0% | 0% |
|  | 3 shots | 0% | 23% | 63% | 0% | 42% |
|  | 4 shots | 77% | 19% | 0% | 0% | 0% |

Supplementary Table 5: Parents were classified into six groups by their vaccination program preferences.

|  |  | Prefer governmental clinics | Careful deciders | Convenience focused | Less co-administration | Cost-sensitive | Middle careful dividers |
| --- | --- | --- | --- | --- | --- | --- | --- |
|  |  | 20% | 21% | 24% | 8% | 15% | 12% |
| Location | Government clinic | 100% | 86% | 3% | 0% | 100% | 0% |
|  | Private clinic | 0% | 14% | 97% | 100% | 0% | 100% |
| Waiting time | 20 min | 86% | 59% | 39% | 0% | 23% | 73% |
|  | 40 min | 0% | 0% | 32% | 100% | 42% | 0% |
|  | 60 min | 0% | 21% | 0% | 0% | 35% | 18% |
|  | 90 min | 14% | 20% | 29% | 0% | 0% | 9% |
| How many visits | 3 visits | 75% | 0% | 79% | 0% | 58% | 0% |
|  | 5 visits | 25% | 0% | 0% | 0% | 42% | 80% |
|  | 7 visits | 0% | 50% | 0% | 100% | 0% | 20% |
|  | 8 visits | 0% | 50% | 21% | 0% | 0% | 0% |
| Cost | 100 RMB | 0% | 0% | 18% | 0% | 100% | 20% |
|  | 200 RMB | 100% | 14% | 0% | 100% | 0% | 0% |
|  | 400 RMB | 0% | 65% | 29% | 0% | 0% | 34% |
|  | 800 RMB | 0% | 21% | 53% | 0% | 0% | 46% |
| Number co-administered injections | 1 shot | 0% | 36% | 0% | 100% | 58% | 46% |
|  | 2 shots | 25% | 21% | 47% | 0% | 0% | 0% |
|  | 3 shots | 0% | 42% | 50% | 0% | 42% | 0% |
|  | 4 shots | 75% | 0% | 3% | 0% | 0% | 54% |
